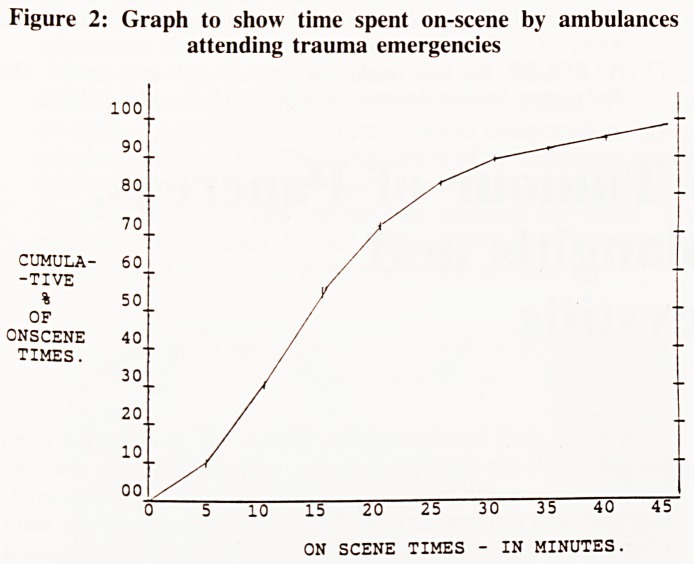# Effect of the Cornwall Helicopter Ambulance on Ambulance Service Emergency Response Time

**Published:** 1991-09

**Authors:** Andrew Rouse

**Affiliations:** Senior Registrar in Public Health Medicine Frenchay Health Authority

**Keywords:** Ambulance, Emergency Medical Services, Emergency Care, Air Ambulance Evaluation

## Abstract

**Objective:**

To determine whether availability of the Cornwall Helicopter Ambulance enabled the ambulance service to arrive more rapidly at the scene of emergencies.

**Design:**

Retrospectively collected ambulance service data analyzed longitudinally.

**Setting:**

The ambulance service in Cornwall.

**Subjects:**

Patients carried as emergencies by the ambulance service.

**Variable Studied:**

Augmentation of a county ambulance service by a Helicopter Ambulance.

**Outcome Measure:**

Ambulance 'response times'.

**Results:**

A small improvement in the ambulance service's overall ability to respond to emergency calls was observed.

**Conclusion:**

Availability of the Helicopter Ambulance marginally improved the ambulance service's response times. It is doubtful if these improved response times were of any clinical significance. More conventional and less expensive means of improving ambulance service performance should be considered by a Health Authority or ambulance service before a helicopter ambulance is deployed.


### Effect of the Cornwall Helicopter Ambulance on Ambulance Service Emergency Response Time

**Andrew Rouse MFPHM**
Senior Registrar in Public Health Medicine
Frenchay Health Authority

**ABSTRACT**

**Objective:** To determine whether availability of the Cornwall Helicopter Ambulance enabled the ambulance service to arrive more rapidly at the scene of emergencies.

**Design:** Retrospectively collected ambulance service data analyzed longitudinally.

**Setting:** The ambulance service in Cornwall.

**Subjects:** Patients carried as emergencies by the ambulance service.

**Variable Studied:** Augmentation of a county ambulance service by a Helicopter Ambulance.

**Outcome Measure:** Ambulance 'response times'.

**Results:** A small improvement in the ambulance service's overall ability to respond to emergency calls was observed.

**Conclusion:** Availability of the Helicopter Ambulance marginally improved the ambulance service's response times. It is doubtful if these improved response times were of any clinical significance. More conventional and less expensive means of improving ambulance service performance should be considered by a Health Authority or ambulance service before a helicopter ambulance is deployed.

**Keywords:** Ambulance Emergency Medical Services Emergency Care Air Ambulance Evaluation.

### INTRODUCTION

**Reason for study.** The study reported here was commissioned by the Cornwall and Isles of Scilly Health Authority (HA). The HA wanted a report on the clinical benefit arising from the operation of the Cornwall Helicopter ambulance in the previous year. The HA was coming under increasing pressure to provide regular funding for the helicopter and understandably wanted a report of its benefit.

### Background

Cornwall's pioneering helicopter ambulance was launched in April 1987: to date it has flown more than 2,000 missions. It is manned by two Cornwall Ambulance Service paramedics. Most emergency patients carried by the ambulance service are brought to Truro hospitals. It is paid for by voluntary subscriptions totalling £30,000 a month. It receives no financial support from the Cornwall and Isles of Scilly Health Authority. Cornwall has a population of over 450,000 and covers over 1,400 square miles. Large areas, particularly in the east of the county, are thinly populated and some residents live over 35 miles from a District General Hospital. The Cornwall Ambulance Service answers over 16,000 emergency calls a year. Day and night about 16 ambulances are on standby throughout the county waiting to attend emergencies. One or more ambulances are dispatched by a controller in Truro Ambulance Service HQ upon receipt of a 999 call. There has been concern that due to the county's size and population scatter the ability of the ambulance service to respond rapidly to emergency calls may be inadequate. Road congestion in summer by thousands of holiday makers might also make the provision of a timely emergency service difficult. One of the reasons that the helicopter ambulance was introduced was to improve the ambulance service's speed of response.[1]

### Need for an helicopter ambulance in Cornwall

The reasons why the Cornwall helicopter ambulance service was initiated are obscure. Certainly the HA and ambulance service have never documented need for such a service.

### Control of helicopter ambulance

The Cornwall Ambulance Service has assumed operational control of the helicopter ambulance. Although some guidelines have been formulated to aid in its deployment it is often used in a very ad-hoc manner. Neither at the inception of the helicopter service or subsequently were specific performance standards or objectives set by the ambulance service or helicopter sponsors. It has operated with only minimal supervision. A very rudimentary (clinically and epidemiologically meaningless) surveillance system is in place.

### Possible benefits of an Helicopter ambulance

A review of the literature and discussion with ambulance personnel suggests eight different mechanisms by which use of an helicopter ambulance might be advantageous.[2, 3, 4] It is convenient to consider these under headings Direct, Indirect, Incidental and Financial advantages, as shown in Table 1.

**Table 1: Possible Advantages of an Air Ambulace**

| |
|---|
| **DIRECT ADVANTAGES** |
| 1. Lifeboat role — it can reach places land ambulances cannot reach. |
| 2. Rapid transport of paramedics to scene. |
| 3. Rapid transport of critically ill to A & E departments. |
| **INDIRECT ADVANTAGES** |
| 4. Effectiveness of the entire ambulance service might be increased. |
| **INCIDENTAL ADVANTAGES** |
| 5. An air ambulance can take critically injured patients to tertiary care centres quickly. |
| 6. It is possible that an air ambulance can reduce "transport trauma". |
| **COST ADVANTAGES** |
| 7. An air ambulance might be able to replace land ambulances with no detriment to the service but more cheaply. |
| 8. If land ambulance fleets are providing unsatisfactory service, the cheapest way to improve the service might be the use of a helicopter ambulance. |

The public and many health service personnel who support the use of the helicopter ambulance generally view it as "a death-defying, life-saving, drop-from-the-sky machine" —

i.e. performing the first three roles indicated in Table 1. However data on file in Cornwall suggests that serious emergencies are few and far between in Cornwall and have been dealt with in a very timely way by the conventional land ambulance service. Senior ambulance officers are aware of this. They feel that the major benefit of the helicopter ambulance is that the entire ambulance fleet responsible for covering emergency calls can be deployed more effectively when the helicopter ambulance is on duty. This is because the helicopter ambulance can provide a much more flexible 'back up cover' to any part of the county temporarily deprived of cover than other land ambulances can. Without the availability of the helicopter ambulance, when a land abulance covering a given area has been deployed, the remaining land ambulances throughout the county have to be moved (often many miles) to different standby locations. This is done so that emergency cover is re-established in that area. Ambulance controllers feel that the availability of the helicopter ambulance can lessen or obviate the need for moving the remaining land ambulances around. This advantage is shown as advantage number 4 in Table 1. It is realization of this advantage which provides the justification for the Ambulance Service's continued deployment of the helicopter ambulance. Ironically the helicopter ambulance can perform this role even if it rarely carries a patient — or indeed even if it never flies. In practice, whilst fulfilling this role it inevitably transports patients with trivial injuries.

### Outcome measure

The only absolute measure for evaluating the effectiveness of any therapeutic intervention is a decrease in morbidity and mortality. It is these which should ideally be studied when the impact of the helicopter is examined. Preliminary studies showed however that serious injury is an infrequent event in Cornwall. Even if the beneficial effect of a helicopter were to reduce mortality by half (from 5% to 2.5%) it would take many years to enrol enough patients in a study to demonstrate convincingly such improvement. It was, therefore, decided to use as a proxy measure of outcome, ambulance "response time". The use of proxy outcome measures is a commonly employed epidemiological technique. The "response time" is the time difference between the ambulance service being notified of an incident (usually by 999 call) and an ambulance arriving at the scene of that incident. Ambulance services throughout England and Wales have been monitoring the 20 minute response times percentage (generally referred to as the ORCON standard) since 1975 when their use was recommended in the ORCON report.[5]

### The ORCON standard

No medical or surgical evidence was considered when this standard was set. The ORCON standard as it applied to Cornwall requires that:—

95% of emergency calls will be reached by ambulance service personnel in 20 minutes.

The rationale of this standard rests on the presumption that useful therapy can be given by ambulancemen when they arrive at scene, at which point the 'therapy free interval' has ended. This standard lacks credibility as a monitoring instrument for several reasons:—

a) The ORCON standard was introduced for administrative convenience. It never had any clinical significance. The predictive value of this standard has never been established.
b) It fails to differentiate between medical, surgical or trauma cases. It is generally agreed[6] that a 20 minute response time for medical emergencies is too great and is of even more doubtful significance in trauma cases where rapid delivery to a major A & E department is all important.
c) Cornwall ambulance service data on file shows that most ambulance service emergency calls are of a trivial nature so it is quite possible that the 95%, 20 minute standard is being achieved overall, but not for the (rarer) more serious cases.
d) The ORCON standard presumes that useful therapy can be and is given by ambulance crews, yet makes no attempt to confirm that such care is given.

Despite its weaknesses ORCON is considered the gold-standard and occasional failure to meet this standard by the Cornwall Ambulance Service is used by the Service as justification for both claims to the District Health Authority for increased funding, and continuing use of the helicopter ambulance.

### Study hypothesis

This study attempts to evaluate whether the benefit of improved performance (advantage 4, Table 1) of the entire emergency ambulance fleet — as measured by the 20 minute response time — occured.

### METHOD

### Formation of study groups

The ambulance service maintains records of the response times to all emergency calls. During the study periods, because of financial constraints, the helicopter ambulance operated for five out of seven days each week. This practice was noted and used in the formation of study groups. The helicopter ambulance always operates in daylight hours. It usually worked either 9 a.m. to 5 p.m. in winter, or 10 a.m. to 6 p.m. in summer months. Missions arising during hours when the helicopter ambulance would not have been scheduled to work (e.g. after 5 p.m. and before 9 a.m. were excluded from study). A comparison was made between response times arising when the helicopter ambulance was on duty (figure 1 group A), and response times arising during comparable daylight hours when the helicopter ambulance was not available (figure 1 group B). The formation of study groups as shown in this way has many analogies with other 'natural experiments'. Natural experiments occur when ".... two or several groups in a population may have different exposures to a factor in their environment".[7]

**Figure 1: Formation of study cohorts**

### Time Period of studies

During the time period of the first study, the 42 weeks following 7 March 1988, missions dispatched by all controllers (either full time or part time controllers) were studied. During the time period of the second study, the 25 week period starting 1 January 1989, missions dispatched by the five full time and presumably more skilful controllers were studied.

### Findings

In the 42 weeks of the first study period, 4,985 responses were made by the ambulance service when the Helicopter Unit was operating, or in comparable daylight hours when it could have been operating. They were activated by full and part-time controllers. The 20 minute response time percentages were improved by 1.4% when the Helicopter Unit was available (Table 2a). This difference of 1.4% at 20 minutes is not statistically significant. (95% Confidence Interval = 0 to 2.8%).

**Table 2a: Completed Response Times in 20 Minutes**

For Full-time and Part Full-time and Part-time Controllers:

| | No. of Missions | No. of Missions with response times 20 mins | Percent complete within 20 minutes |
|---|---|---|---|
| Dispatched when helicopter available | 3504 | 3342 | 95.4 |
| Dispatched (similar hours) when helicopter not available | 1481 | 1393 | 94.0 |
| **Total** | 4985 | 4735 | |
| | | | Range 1.4% |

**Table 2b: Completed Response Times in 20 Minutes**

For five Full-time Controllers:

| | No. of Missions | No. of Missions with response times 20 mins | Percent complete within 20 minutes |
|---|---|---|---|
| Dispatched when helicopter available | 1513 | 1450 | 95.8 |
| Dispatched (similar hours) when helicopter not available | 692 | 642 | 92.0 |
| **Total** | 2205 | 2092 | |
| | | | Range 3.0% |

In the 25 weeks of the second study period, 2,205 missions were dispatched in comparable daylight hours by full-time controllers. The 20 minute response time percentages were improved by 3% when the Helicopter Unit was available (Table 2b). The difference of 3.0% at 20 minutes is statistically significant (95% Confidence Interval = 0.8% to 5.2%).

### DISCUSSION

### Selection bias

The five days when the helicopter ambulance was operating were not selected randomly by the ambulance service. The service made deliberate attempts to redeploy the helicopter ambulance on days which were likely to be busy. However, except that the helicopter ambulance invariably worked on Mondays (Bank holidays are invariably Mondays), review of ambulance service records revealed little to suggest that days when helicopter ambulance was deployed were different from days when it was not employed.

### Formation of study groups

The best way to form study cohorts would be to randomly deploy the helicopter to support the land ambulance service some days and not on others. However reliance had to be placed on previously collected data. Randomization cannot be done retrospectively.

### Effect of helicopter ambulance on response times

The use of a helicopter ambulance was associated with slight improvements in the 20 minute response percentages sufficient to achieve the ORCON 20 minute standard. This is considered important by the ambulance service. However as discussed previously it is doubtful if this was of any benefit to patients.

### The value of alternative interventions

The slight improvements in response times are not surprising. Presumably response times can be improved by numerous other interventions. A review of the literature and discussions with ambulance service personnel suggests that ambulance services appear not to have considered assessing the marginal benefits and costs from deploying extra land ambulances or the introduction of other changes. Therefore it is uncertain whether the improvements in response times brought about by the introduction of the Helicopter Ambulance are greater or lesser than those which could have been brought about in more conventional ways. The following two vignettes may however put this Helicopter associated 1-3% improvement in the ORCON 20 minute standard in slightly clearer perspective.

### Vignette 1 - Controller Expertise

Observation of ambulance service operations and discussion with senior ambulance service staff suggest that it is the actions of the duty controller which determines the effectiveness of the emergency ambulance service. However there is no monitoring system in place which can evaluate the performance of these controllers. By using a similar methodology to that described above it was possible to prepare quarterly ORCON performance figures for each controller. The results for the first three quarters of 1989 are shown in table 3.

**Table 3:** Differences in the percentage of patients attended in 20 minutes for the five **Principal Ambulance Controllers**

| | MONITORING PERIODS | | |
|---|---|---|---|
| | January February March | April May June | July August September |
| Controller with best performance | A 96.5% | D 93.4% | E 92.1% |
| Controller with 2nd best performance | B 94.6% | A 93.3% | D 91.7% |
| Controller with 3rd best performance | C 94.0% | B 92.9% | B 91.3% |
| Controller with 4th best performance | D 93.8% | C 91.0% | A 90.7% |
| Controller with worst performance | E 92.8% | E 90.4% | C 87.9% |
| | Range 3.7% | Range 3.0% | Range 4.2% |
| Total Number of Missions | 2273 | 3162 | 3795 |

Controllers identified anonymously as A, B, C, D and E.

A 3-5% difference in performance is noted consistently between the best and worst controller. This is similar to the 1.4% or 3.0% improvement (table 2) associated with the use of the Helicopter Unit. The results have some correlation with staff recruitment and morale. For instance, controller D was new to the job in the spring 1989 and had poorer performance in that quarter than subsequently. It is likely that this improved response reflects his learning and acquiring experience. Controller E is normally a competent experienced employee whose general performance had deteriorated. When he was counselled in June his subsequent performance improved. What is clear is that the 2-3% improvement associated with the use of the Helicopter Unit is of the same order as might be expected if a training and monitoring scheme were introduced with the aim of improving controller performance. It is very likely that such a training and monitoring program would cost considerably less than the £360,000 a year it costs to run the helicopter ambulance.

**Vignetter 2 — Monitoring on-scene times**
The ambulance service records of 737 cases taken to the City Hospital Truro were reviewed and a note made of on-scene times. Surprisingly on-scene times were more than 15 minutes in over 50% of missions! These findings are shown in figure 2.

**Figure 2: Graph to show time spent on-scene by ambulances attending trauma emergencies**

**Data obtained from 737 Emergency trauma missions taken to the Royal Cornwall Hospital, Truro**
These results were discussed with senior ambulance service officers, who are in agreement that in many instances far too long is being spent on scene by ambulance crew. The Chief Ambulance officer feels that most on-scene times should be less than 10 minutes. Since, when an ambulance is at the scene of an incident it is unavailable to respond to other calls, it is likely that a substantial increase in ambulance availability would occur if ambulances spent shorter times on-scene. It can be calculated that if all on-scene times were reduced to 10 minutes this would be equivalent to a 2% increase in ambulances on duty. It is quite possible that a 2% increase in the functional level of ambulances on duty could be associated with an improvement in the ORCON standard by 2-3%, i.e. an amount comparable to that observed when the Helicopter Unit is on duty. It should not be difficult or expensive to alter operational procedures to ensure that these shorter on-scene times are achieved.

**Extrapolation of findings**
This study dealt with the helicopter ambulance functioning in only one of the 8 possible roles (role 4) shown in table 1. The observed improved performance brought about by the helicopter was so marginal and of such doubtful clinical significance that it cannot provide adequate justification for the HA to financially support the helicopter ambulance. It is important to note that it was senior ambulance staff — who incidentally are firm believers in the helicopter's effectiveness — who suggested that it should be "role 4" (and not any of the 7 other roles) of the helicopter ambulance which should be evaluated. They suggested this because they felt strongly that it was in fulfilling this role that benefit of the helicopter ambulance would most likely be observed. This being the case it is unlikely that a case can be made for the HA supporting the air ambulance whilst it performs it's 7 other roles since it is likely that the value of the helicopter in performing its other possible roles would be even less than those described here!

**Is there a role for a helicopter ambulance in Cornwall —**
Serious emergencies are few and far between in Cornwall and are generally attended to very quickly by the conventional land ambulance service. For instance an Office of Population Census and Survey report for 1988 shows 150 deaths due to injury occurred in residents of Cornwall.[8] A report by the Royal College of Surgeons suggests that about half of these deaths would have occurred within a few minutes of being injured and are therefore inevitable.[9] The same report also suggests that 13% of the remainder of these deaths (1 a month) might under the most favourable circumstances be preventable. Since the Cornwall Helicopter ambulance is only operational 33% of the time (seven 8 hour shifts a week), only about once every 3 months will the Helicopter be operational when a traumatic incident occurs in which a patient is potentially salvageable. Quite clearly the occasions when an helicopter ambulance can "death defy and drop from the sky" are infrequent.

**— and can the NHS in Cornwall afford it?**
The Cornwall Helicopter ambulance costs about £1,000 a day to operate. Making the optimistic assumption that availability of the Helicopter ambulance alone can result in the saving of all these lives (4 persons per year), and that similar numbers of very seriously injured persons are returned to full health (another 4 persons per year) as a result of the availability of an helicopter ambulance, we can calculate that each life saved or serious disability averted costs £350,000/8, or £44,000 per life. Some authorities have suggested that the NHS cannot afford to spend more than £14,000 (1982 pounds) per potential life saved.[10] This is equivalent to £19,800 contemporary pounds. If this view is held it would appear that there can be little justification for the NHS investing in Helicopter ambulances.

**Advice to other Health Authorities considering setting up an helicopter ambulance service**
Experience gained whilst performing this and other studies reported elsewhere[11,12] enables me to make the following recommendations.

It is very important that a helicopter ambulance service should not be commissioned until:—

1. A needs assessment has been carried out.
2. Deficiencies are identified that are potentially remediable by use of an helicopter ambulance.
3. Clinically relevant performance standards with specific quantitative and qualitative specifications should be set **prior to deployment.**
4. A helipad is provided in close proximity to the A&E department.

A needs assessment, identifying deficiencies, setting performance standards and monitoring performance will require the development of an Information System. Details of such a system are discussed elsewhere.[13] Establishment of a suitable Information System would take at least one year. Collecting and analyzing data would take a further 12 months. Therefore even if ambulance services initiate the development of an information system now, rational and effective deployment of a helicopter ambulance should not be contemplated for at least another 2 years.

**Action arising**
Partly as a result of this study, the HA has chosen not to fund the helicopter ambulance service.

**PS. Follow up July 1991**
The helicopter is still operating. There is still no means of effectively monitoring its deployment.

### CONCLUSION
The helicopter ambulance improved the ambulance service response times to a slight and clinically unimportant degree. The Cornwall Health Authority is correct in not providing financial support for the helicopter ambulance. Before other Health Authorities consider commissioning a helicopter ambulance attempts should be made to identify weaknesses in service which are remediable by more conventional and less expensive means.

### ACKNOWLEDGEMENTS
Thanks to M. Sheen, D. Miles, G. Evans, W. Poulsom, W. Moore.

### REFERENCES
1. First air ambulance — WHY? Information leaflet released by First Air Ambulance Service Trust. Tregeare, West Down, Delabole, Cornwall.
2. Helicopter Emergency Medical Services. Unsigned editorial report. College and Faculty Bulletin Supplement to the Annals of the Royal College of Surgeons of England. 1989; 71(4): 60-3.
3. H. CHAMPION. Organization of trauma care. *Trauma management,* Eds D. Kreis, G. Gomez. Little Brown & Company 1989.
4. R. A. ALEXANDER. Organization of trauma care. *Trauma management,* Eds. D. Kreis, G. Gomez. Little Brown & Company 1989.
5. J. A. BARNES, J. R. MERCHANT, N. WEBSTER. The Ambulance Service; performance, standards and measurement. The Ambulance Operational Research Unit, Cranfield Institute of Technology, 1974.
6. REDMOND A. D. Paramedics in the United Kingdom. *British Medical Journal,* 1984; **288:** 622-623.
7. Pekka Puska in 'Intervention and experimental studies'. *Oxford Textbook of Public Health,* Eds W. W. Holland, R. Detels & G. Knox. *Oxford University Press,* 1985:*(3)* 113. 19857.
8. VS3 — Mortality statistics, Cornwall and Isles of Scilly District Health Authority 1988. OPCS.
9. Report of the Working Party on the management of Patients with major Trauma. *Royal College of Surgeons,* November 1988, page 16.
10. ROBERTS C. J., FARROW S. C. and CHARNEY M. C. How much can the NHS afford to save a life or avoid a severe disability? *The Lancet.* 1985;**i**: 89-91.
11. A. ROUSE. Study to examine the timeliness of care received by patients with open fractures of the lower limb. To appear Journal of Public Health Medicine.
12. A. ROUSE. The effect of the Cornwall & Isles of Scilly Helicopter Ambulance Unit on the ambulance service's ability to deliver patients to an Accident & Emergency Department. Accepted Archives of Emergency Medicine.
13. A. ROUSE. So you think you need an Air-ambulance? *The Ambulance Service Journal.* Vol 20:**2**; 16-17, March 1991.

## Figures and Tables

**Figure 1 f1:**
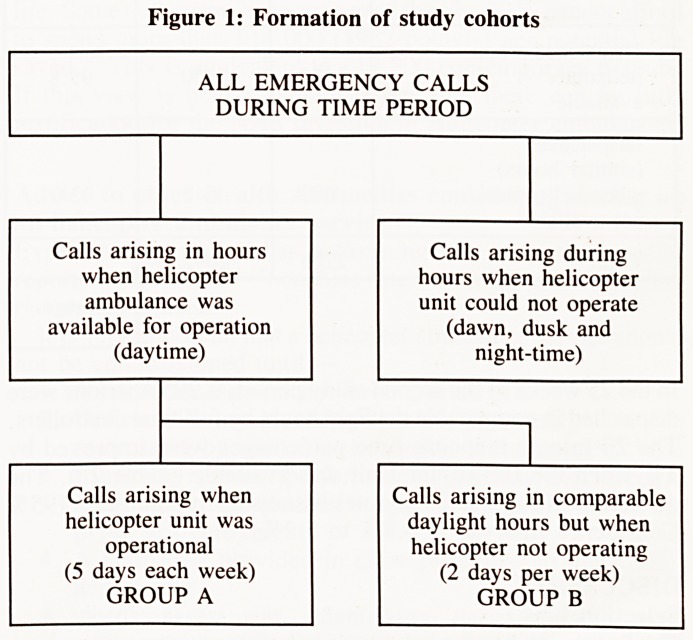


**Figure 2 f2:**